# Comparison of the structural, electrochemical, and spectroscopic properties of two cryptates of trivalent uranium†

**DOI:** 10.1039/d4dt00521j

**Published:** 2024-05-02

**Authors:** D. Nuwangi Kulasekara, Matthew D. Bailey, Cassandra L. Ward, Matthew J. Allen

**Affiliations:** a Department of Chemistry, Wayne State University 5101 Cass Avenue Detroit MI 48202 USA mallen@chem.wayne.edu; b Lumigen Instrument Center, Wayne State University 5101 Cass Avenue Detroit MI 48202 USA

## Abstract

We describe a study of the influence of cryptand denticity on the structural, electronic, and electrochemical properties of U^III^-containing cryptates. Two cryptands (2.2.2 and 2.2.1) are reported. The cryptand with the smaller denticity leads to negative electrochemical potentials and shorter bond lengths that are consistent with a better fit for U^III^ than the larger cryptand. These studies provide insight into the rational design of cryptand-based ligands for trivalent uranium.

## Abstract 2

Two cryptates of trivalent uranium are compared with respect to luminescence, solid-state structure, and electrochemistry.

## Graphical abstract

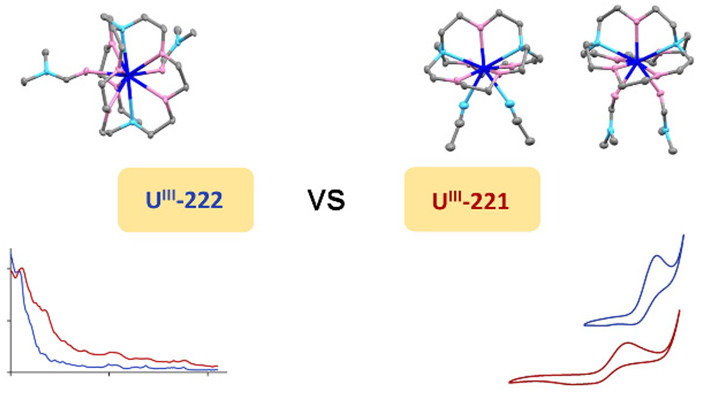

## Introduction

**Fig. 1:**
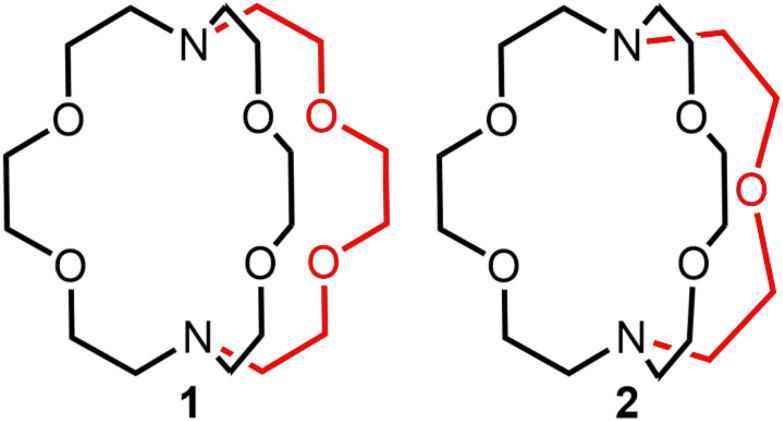
2.2.2-Cryptand, 1, and 2.2.1-cryptand, 2, that were studied with U^III^. The red color highlights the difference between the two ligands.

The accumulation of depleted uranium as a waste product from uranium enrichment encourages the development of research focused on uranium coordination chemistry.^1–3^ Reported studies in this area provide insight into potential uses of depleted uranium and fundamental knowledge of uranium coordination chemistry involved in applications such as actinide separations in nuclear waste.^4–8^ Within this context, there have been widespread reports of redox-active and redox-inactive ligands used to form complexes of U^III^,^9–16^ and one of those ligands, 2.2.2-cryptand, has been widely used to encapsulate metal ions, including U^III^.^17–19^ Further, the coordination chemistry of U^III^, Np^III^, and Pu^III^ with 2.2.2-cryptand has been reported recently,^20^ expanding cryptand chemistry into the actinides. The thermodynamic and kinetic stability of a cryptate is governed by the cavity size and denticity of the coordinated cryptand as well as the ionic radii and oxidation state of the given metal ion.^21–24^ For example, 2.2.1-cryptand fits better with Eu^III^, and 2.2.2-cryptand fits better with Eu^II^; moreover, the Gibbs free energy of Eu^III^(2.2.1-cryptand) is 1.8 times greater than that of Eu^III^(2.2.2-cryptand), and the dissociation constant of Eu^III^(2.2.1-cryptand) is 2.7 × 10^3^ times smaller than that of Eu^III^(2.2.2-cryptand).^24^ Because the size of U^III^ (1.025 Å) is closer to the size of Eu^III^ (0.947 Å) than Eu^II^ (1.17 Å)^25^ and the charge density of U^III^ is closer to Eu^III^ than Eu^II^, we suspected that 2.2.1-cryptand would be a good ligand for U^III^. Additional support for this suspicion is in our recent report that the flexible counterpart of 2.2.2-cryptand, tris[2-(2-methoxyethoxy)ethyl]amine (TDA-1), forms U^III^-containing complexes with smaller coordination numbers (nine) compared to all reported 2.2.2-cryptates (with coordination numbers of ten).^26^ This report of an acyclic ligand implies that trivalent uranium can be encapsulated by cryptands with smaller denticities than that of 2.2.2-cryptand. Therefore, based on the studies of Eu^III^ cryptand chemistry and U^III^ chemistry with acyclic TDA-1, we hypothesized that 2.2.1-cryptand is a better match for U^III^ than 2.2.2-cryptand. Here, we report U^III^-containing cryptates of 2.2.2- and 2.2.1-cryptand (Fig. 1) to investigate how ligand denticity affects the structural, spectroscopic, and electrochemical properties of U^III^.

## Results and discussion

**Scheme 1:**
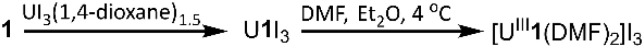
Synthesis of U^III^-containing complex of 1.

**Scheme 2:**
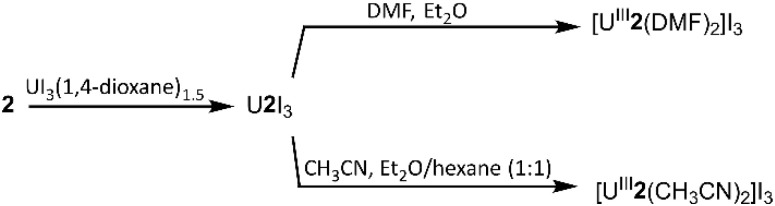
Synthesis of U^III^-containing complexes of 2.

**Fig. 2:**
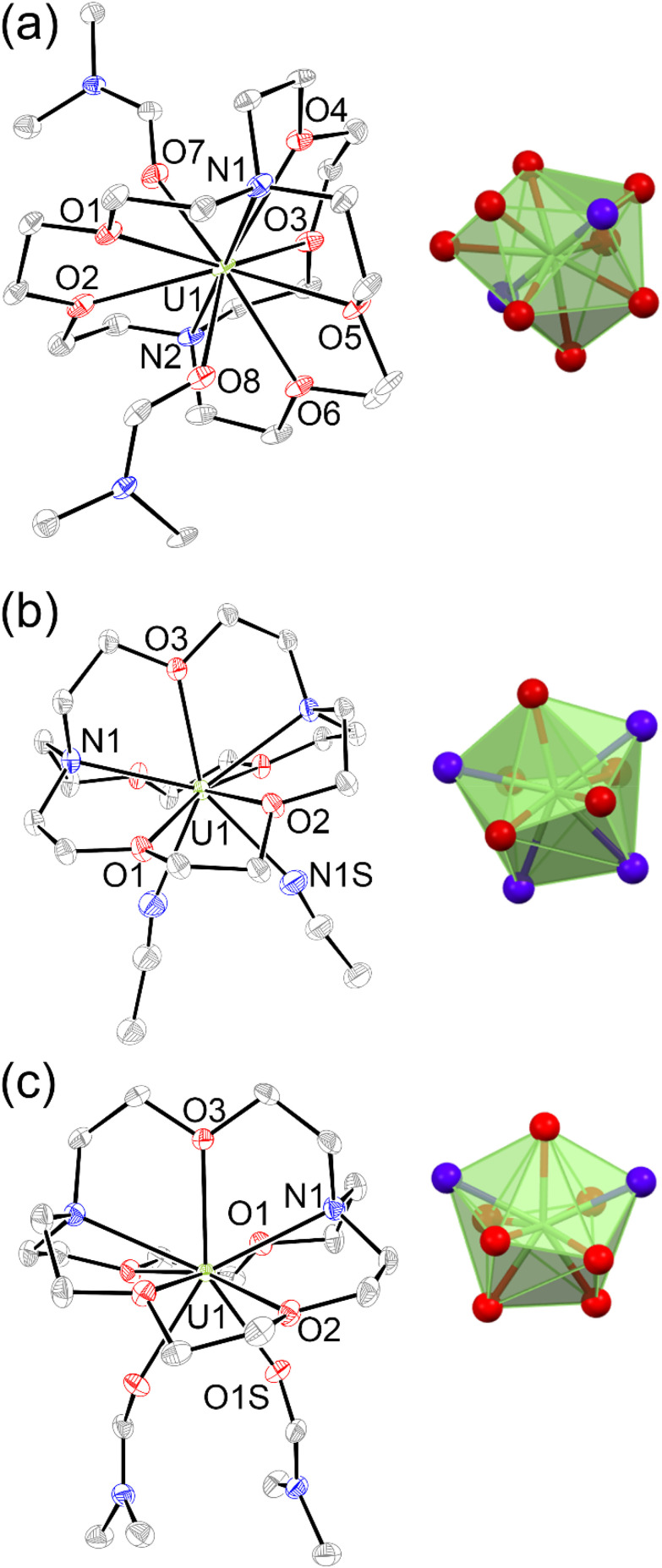
Molecular structures in crystals of (a) [U^III^1(DMF)_2_]I_3_, (b) [U^III^2(CH_3_CN)_2_]I_3_, and (c) [U^III^2(DMF)_2_]I_3_. Thermal ellipsoids are drawn at 50% probability. Hydrogen atoms and iodide counter ions are omitted for clarity. Blue = nitrogen; red = oxygen; gray = carbon; and green = uranium. Crystallographic data for these structures have been deposited at the Cambridge Crystallographic Data Centre under deposition numbers CCDC (2248985–2248987†).

To evaluate the structural properties of U^III^-containing cryptates, crystals were grown from *N*,*N*-dimethylformamide (DMF) or acetonitrile (CH_3_CN) (Schemes 1 and 2), and the structures of [U^III^1(DMF)_2_]I_3_, [U^III^2(CH_3_CN)_2_]I_3_, and [U^III^2(DMF)_2_]I_3_ were solved from the crystals (Fig. 2). All three complexes contained coordinated solvent molecules. The structure with 1 is like reported structures of 2.2.2-cryptates of trivalent uranium that share ten-coordinate structures with bound solvent molecules, iodide,^18,20^ triflate,^22^ or water molecules.^18,20^ The geometry of [U^III^1(DMF)_2_]I_3_, analyzed by SHAPE (v.2.1),^27^ is sphenocorona. We also performed SHAPE analysis for the reported structure of [U^III^1I(CH_3_CN)]I_2_ with one inner-sphere iodide and one inner-sphere molecule of CH_3_CN (CCDC number 2020050),^19^ and we found that it also has the sphenocorona geometry. The crystal structure that we report here contains two coordinated molecules of DMF instead of iodide, water, or triflate, but the change in monodentate donors does not change the geometry about U^III^. In contrast to [U^III^1(DMF)_2_]I, U^III^ with 2 adopts a nine-coordinate structure with two molecules of CH_3_CN {[U^III^2(CH_3_CN)_2_]I_3_} or two molecules of DMF {[U^III^2(DMF)_2_]I_3_} coordinated to the opposite face of uranium relative to the single-oxygen-bearing arm of 2. Both [U^III^2(CH_3_CN)_2_]I_3_ and [U^III^2(DMF)_2_]I_3_ possess spherical-relaxed capped cube geometries as determined by SHAPE (v.2.1). U^III^-containing complexes of 1 and 2 exhibit poor solubility in ethereal and nonpolar solvents, and for this reason, DMF and CH_3_CN were used for crystallization. Comparison of bond lengths between [U^III^2(CH_3_CN)_2_]I_3_ and [U^III^2(DMF)_2_]I_3_ reveals no noticeable variation in bond lengths between uranium and the donor atoms in 2; however, the U–O_DMF_ bond in the complex with coordinated DMF is shorter than the U–N_CH_3_CN_ bond in the complex with coordinated CH_3_CN, as expected based on the difference in size between O and N (Table 1). Similarly, U–O_ether_ bonds in [U^III^2(CH_3_CN)_2_]I_3_, [U^III^2(DMF)_2_]I_3_, and [U^III^1(DMF)_2_]I_3_ are shorter than U–N_amine_ bonds. The complexes [U^III^2(CH_3_CN)_2_]I_3_ and [U^III^2(DMF)_2_]I_3_ each contain U–O_ether_ and U–N_amine_ bonds that are 0.1–0.2 Å shorter than similar bonds in [U^III^1(DMF)_2_]I_3_. However, U–O_DMF_ bond lengths in [U^III^1(DMF)_2_]I_3_ and [U^III^2(DMF)_2_]I_3_ do not exhibit large differences relative to each other. The U–O_ether_ and U–N_amine_ bonds in [U^III^2(CH_3_CN)_2_]I_3_ and [U^III^2(DMF)_2_]I_3_ are shorter than analogous bonds in reported U^III^-containing complexes with TDA-1.^26^

**Table 1:** Average bond lengths of U^III^1, U^III^2, and U^III^(TDA-1)

	Average U–donor distances (Å)			
Complex	U–O_(ether)_	U–N_(amine)_	U–I	U–S_(solvent)_
[U^III^1(DMF)_2_]I_3_	2.656(5)	2.790(6)	NA	2.434(6)
[U^III^2(DMF)_2_]I_3_	2.547(3)	2.683(3)	NA	2.471(3)
[U^III^2(CH_3_CN)_2_]I_3_	2.507(3)	2.661(3)	NA	2.644(4)
[U^III^1(I)(CH_3_CN)]I_2_ ^19^	2.640(2)	2.818(3)	3.2594(7)	2.647(3)
[U^III^(TDA-1)(I)_2_]I ^26^	2.605(6)	2.767(7)	3.2045(7)	NA

**Fig. 3:**
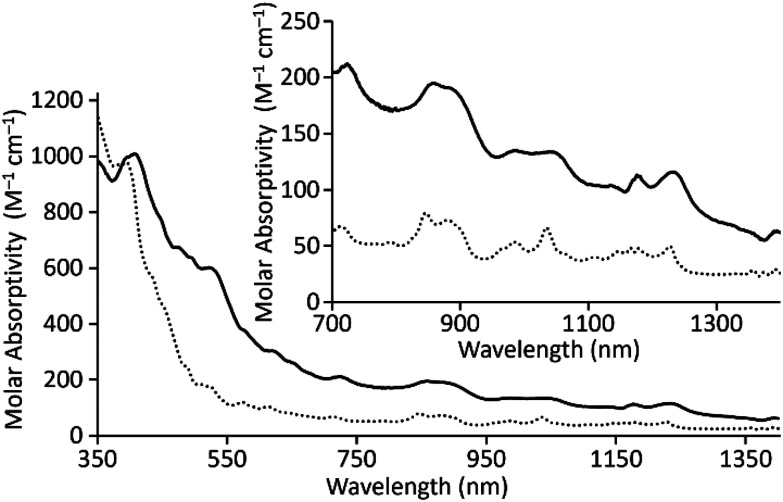
Electronic absorption spectra for U^III^1I_3_ (⋯) and U^III^2I_3_ (—) in CH_3_CN.

In addition to solid-state characterization, electronic spectroscopic characterization was performed for U1I_3_ and U2I_3_ to analyze how ligand denticity influences solution properties of the complexes. We performed UV-visible and near-IR experiments using elementally pure U^III^-containing complexes of 1 and 2 that does not include coordinated solvent molecules. Those complexes are referred to as U1I_3_ and U2I_3_. UV-visible and near-IR electronic absorption data were collected from 350 to 1400 nm in CH_3_CN (Fig. 3). In the visible region, color-producing bands in U1I_3_ appeared for the green-yellow solutions in acetonitrile with maximum absorbances at 395 nm (*ε* = 978 M^−1^ cm^−1^). U2I_3_ forms reddish-pink solutions in CH_3_CN with color-producing bands having maxima at 407 nm (*ε* = 1008 M^−1^ cm^−1^) with shoulders at 521 nm (*ε* = 603 M^−1^ cm^−1^). Weak bands appear in the near-IR region for U1I_3_ and U2I_3_ like the other complexes of trivalent uranium with 5f^3^ electronic configurations with Laporte-forbidden f–f transitions.^18,28,29^ These bands in near IR-region are broader than the UI_3_ bands in acetonitrile (Fig. S3†) and the reported acyclic U^III^TDA-1.^26^ The differences in the optical properties of U^III^1I_3_ and U^III^2I_3_ in solution, specifically the large differences in shifts of color producing bands, indicate an influence of the change of ligand denticity from octadentate to heptadentate on 5f–6d orbital energy gaps.

**Fig. 4:**
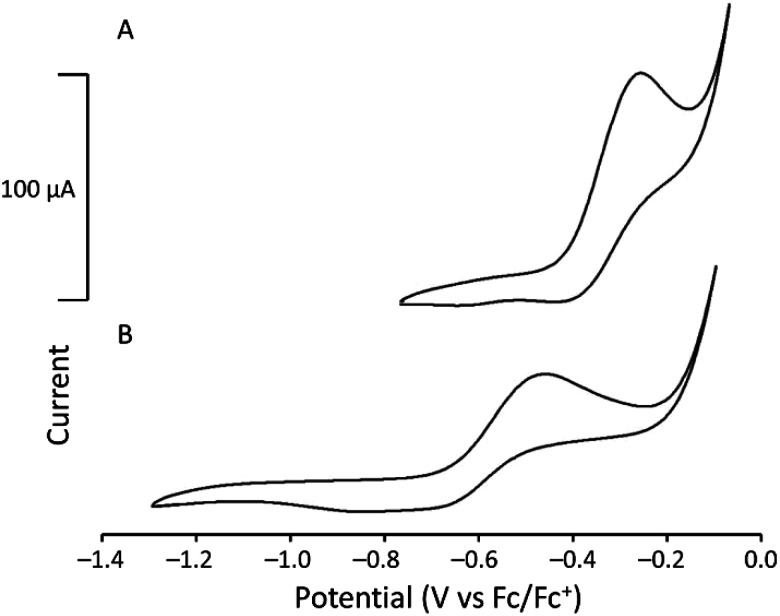
Cyclic voltammograms of (A) [U^III^1]I_3_ (3.36 mM) and (B) [U^III^2]I_3_ (3.42 mM) in CH_3_CN (scan rate = 100 mV s^−1^).

To study the electrochemical behavior of U^III^-containing cryptates, cyclic voltammetry was performed for U1I_3_ and U2I_3_ in CH_3_CN (Fig. 4). Oxidation peaks corresponding to the U^III/IV^ couple appear at −0.26 V *versus* ferrocene/ferrocenium (Fc/Fc^+^) for U1I_3_. The oxidation potential of the U^III/IV^ couple of U1I_3_ in CH_3_CN is like the reported oxidation potential (−0.31 V *versus* Fc/Fc^+^) of U^III^2.2.2-cryptate.^19^ The cyclic voltammogram of U2I_3_ contains an oxidation peak corresponding to the U^III/IV^ couple at −0.46 V *versus* Fc/Fc^+^. Both U1I_3_ and U2I_3_ contain an oxidation peak corresponding to a U^III^-to-U^IV^ oxidation that is not observed in the voltammogram of UI_3_ (Fig. S2†). Cyclic voltammograms of U1I_3_ and U2I_3_ were performed using elementally pure powdered compounds that do not contain coordinated solvent molecules. Both U1I_3_ and U2I_3_ can coordinate at least one acetonitrile molecules in that solid state as evidenced by reported crystal structures^19^ and this study. Similarly, in solution, acetonitrile can coordinate to U1I_3_, U2I_3_, and UI_3_. However, cyclic voltammetry of U1I_3_, U2I_3_, and UI_3_ in this study were performed in the same solvent, acetonitrile; consequently, the variability in shifts of oxidation potentials that arises from the coordination of solvent is minimized. Therefore, the observed 0.2 V difference in oxidation potentials between U1I_3_ and U2I_3_ in acetonitrile most likely arises from changes in the ionization energies resulting from changes in the ligand structure. These results indicate that oxidation potentials of U2I_3_ shift to more negative potentials with the decrease of coordination number compared to the octadentate cryptand in U1I_3_. The negative shift in oxidation potential is likely due to the smaller denticity of 2 compared to 1 and consequent size match between U^III^ and 2. A similar relationship is observed in the reported study between Eu^III^-containing complexes of 1 and 2.^30^ The formal potential of Eu^III^2 (−425 mV *versus* saturated calomel electrode) is more negative than that of Eu^III^1 (−225 mV *versus* saturated calomel electrode). Therefore, the negative shift in cyclic voltammetry of U2I_3_ is consistent with the electrochemistry of 4f systems. Interestingly, U1I_3_ and U2I_3_ have electrochemical potentials among the most positive of reported U^III/IV^ couples of monometallic complexes of uranium.^31^ The reported U^III^ complexes that coordinated to negatively charged donor atoms increase the electron density of uranium and consequently result in more negative electrochemical potentials.^31^ Therefore, the observed positive shift in electrochemical potential is not surprising for U^III^-containing cryptates of 1 and 2 when compared to complexes of cyclopentadienyls, bis(trimethylsilyl)amides, tris(aryloxides), and β-diketiminates.^31^ However, to the best of our knowledge, the relationship of cryptand denticity on electrochemical studies of actinide series has not been reported; therefore, the insight gained from the electrochemical studies described here provides information regarding the influence of cryptand denticity on the tuning of the electrochemical potential of U^III^.

## Experimental

### General methods

All air- and moisture-sensitive reactions were performed using standard Schlenk technique with Ar or using an inert atmosphere dry glove box under an atmosphere of N_2_. Commercially available anhydrous solvents were used for air- and moisture-sensitive reactions after degassing under reduced pressure and drying over activated molecular sieves (CH_3_CN with 3 Å sieves, diethyl ether and DMF with 4 Å sieves, and tetrahydrofuran and 1,4-dioxane with 5 Å sieves).

Depleted uranium was purchased from Manufacturing Science Cooperation (Oak Ridge, TN) and purified following a reported procedure.^32^ UI_3_(1,4-dioxane)_1.5_ was synthesized following a reported procedure.^32^ 4,7,13,16,21,24-Hexaoxa-1,10-diazabicyclo[8.8.8]hexacosane (1) and 4,7,13,16,21-pentaoxa-1,10-diazabicyclo[8.8.5]tricosane (2) were purchased from commercial sources.

Elemental analyses were performed by the Microanalytical Facility at the University of California, Berkeley. Electronic absorption spectra were collected using a Jasco UV-Vis-NIR spectrophotometer, and metal concentrations were determined using energy-dispersive X-ray fluorescence spectroscopy with a Shimadzu EDX-7000 spectrometer at the Lumigen Instrument Center in the Department of Chemistry at Wayne State University.

Cyclic voltammetry was performed using a three-electrode setup with a Pine Wavenow USB potentiostat under an atmosphere of Ar with a glassy carbon working electrode, a freshly prepared Ag/AgCl wire pseudo reference electrode (Ag wire with AgCl coating was prepared by dipping a polished Ag wire in bleach for 5–10 min), and a Pt-wire auxiliary electrode. Acquisition parameters of [U^III^1]I_3_ and [U^III^2]I_3_ were eight segments, an initial potential of −0.5 or 0.0 V (rising), an upper potential of 0.7 V, a lower potential of −0.5 V or 0.0 V against Ag/AgCl pseudo reference electrode, and a scan rate of 100 mV s^−1^. Tetrabutylammonium trifluoromethanesulfonate (0.1 M) was used as the electrolyte, and analyte concentrations were 3.0–3.5 mM. All cyclic voltammograms were recorded in CH_3_CN referenced to an internal standard of Fc/Fc^+^.

A crystal of [U^III^1(DMF)_2_]I_3_ was mounted on a Bruker X8 APEX-II diffractometer with Mo radiation and a graphite monochromator. The X-ray diffraction intensities were measured using a Bruker APEX-II charge-couple device detector. Crystals of [U^III^2(DMF)_2_]I_3_ and [U^III^2(CH_3_CN)_2_]I_3_ were mounted on a Bruker D8 Venture diffractometer with kappa geometry, an Incoatec IμS micro-focus source X-ray tube (Mo Kα radiation), and a multilayer mirror for monochromatization. The X-ray diffraction intensities were measured using a Photon III CPAD area detector. Data for [U^III^1(DMF)_2_]I_3_, [U^III^2(DMF)_2_]I_3_, and [U^III^2(CH_3_CN)_2_]I_3_ were acquired at 100 K with an Oxford 800 Cryostream low-temperature apparatus. The intensities were integrated using SAINT V8.38a software. A multiscan absorption correction was applied with SADABS v2016/2 using APEX4 v2021.10-0. Crystal structures were solved using a dual-space approach as implemented in SHELXT program^33^ and difference Fourier maps during least-squares refinement, as embedded in SHELXL-2018 ^34^ running under Olex2.^35^

### Synthesis of U^III^-containing cryptates

#### U^III^1I_3_ (Scheme 1)

A solution of UI_3_(1,4-dioxane)_1.5_ (193.7 mg, 0.2580 mmol) in tetrahydrofuran (2 mL) was added to a solution of 1 (97.1 mg, 0.258 mmol) in tetrahydrofuran (2 mL). The resulting dark-brown mixture was stirred for 1 h. The precipitate was collected by filtration and washed with tetrahydrofuran (4 × 4 mL). Residual solvent was removed under reduced pressure to obtain U^III^1I_3_ as a dark-brown powder (139.1 mg, 56% yield). Anal. calcd for UC_18_H_36_I_3_N_2_O_6_: C, 21.72; H, 3.65; N, 2.81. Found (%): C, 21.88; H, 3.45; N, 2.63. To prepare crystals, U^III^1I_3_ powder (∼20 mg) was dissolved in DMF (∼1 mL). Vapor diffusion of diethyl ether into the DMF solution at 4 °C yielded green, X-ray quality crystals of [U^III^1(DMF)_2_]I_3_.

#### U^III^2I_3_ (Scheme 2)

A solution of UI_3_(1,4-dioxane)_1.5_ (225.3 mg, 0.3000 mmol) in tetrahydrofuran (2 mL) was added to a solution of 2 (100.0 mg, 0.3000 mmol) in tetrahydrofuran (2 mL). A red precipitate formed, and the reaction mixture was stirred for 1 h. The precipitate was collected by filtration and washed with tetrahydrofuran (4 × 4 mL). Residual solvent was removed under reduced pressure to obtain U^III^2I_3_ as a pink-red powder (70.3 mg, 51% yield). Anal. calcd for UC_16_H_32_I_3_N_2_O_5_: C, 20.20; H, 3.39; N, 2.95. Found (%): C, 19.68; H, 3.01; N, 2.69. Red crystals of [U^III^2(DMF)_2_]I_3_ were formed at ambient temperature by vapor diffusion of diethyl ether into a solution of U^III^2I_3_ (∼20 mg) in DMF (∼1 mL). Red crystals of [U^III^2(CH_3_CN)_2_]I_3_ were formed by vapor diffusion of diethyl ether/hexanes (1 : 1) into a solution of U^III^2I_3_ (∼20 mg) in CH_3_CN (∼1.5 mL).

## Conclusions

This study reports differences in the structural, spectroscopic, and electrochemical properties of U^III^ encapsulated into neutral, redox-inactive cryptands. The smaller denticity and cavity size of heptadentate 2 enabled greater bonding interactions with U^III^ compared to octadentate 1. This evidence of a favorable size match between 2.2.1-cryptand and U^III^ is supported by shorter bond lengths and negative shifts of the electrochemical potentials of U^III^2 compared to U^III^1. These findings provide valuable insight into the encapsulation of trivalent uranium that has potential use areas such as actinide separations relevant to the management of radioactive waste.

## Conflicts of interest

There are no conflicts to declare.

## Supplementary Material

DT-053-D4DT00521J-s001

DT-053-D4DT00521J-s002
