# Supplementary material for: Comparison of the structural, electrochemical, and spectroscopic properties of two cryptates of trivalent uranium
Source: Dalton Trans. 2024 May 2;53(20):8657–61. doi: 10.1039/d4dt00521j (PMC11106807; doi:10.1039/d4dt00521j)
Supplement: DT-053-D4DT00521J-s001 [file DT-053-D4DT00521J-s001.pdf]

Electronic Supplementary Material (ESI) for Dalton Transactions.
This journal is © The Royal Society of Chemistry 2024

Comparison of the structural, electrochemical, and spectroscopic properties of two cryptates of trivalent uranium

D. Nuwangi Kulasekara,[a] Matthew D. Bailey,[a] Cassandra L. Ward,[b] and Matthew J. Allen*,[a]

[a]Department of Chemistry, Wayne State University, 5101 Cass Avenue, Detroit, Michigan 48202, United States
[b]Lumigen Instrument Center, Wayne State University, 5101 Cass Avenue, Detroit, Michigan 48202, United States

*Email: mallen@chem.wayne.edu

Page	Contents
S1	Table of contents
S2	Crystallographic data
S5	*SHAPE* analysis
S7	Cyclic voltammetry
S8	Electronic absorption spectroscopy

Crystallographic data

Table S1. Crystallographic properties of $[U^{III}\mathbf{1}(DMF)_2]I_3$

Chemical formula	$C_{24}H_{50.01}I_3N_4O_8U$	
Formula weight	1141.44	
R_1	0.0367	
wR_2	0.0900	
Goodness of Fit	1.202	
Largest residual peak/hole	0.96/−1.78	
Temperature	100 K	
Wavelength	0.71073 Å	
Crystal system	monoclinic	
Space group	$P2_1/c$	
Unit cell dimensions	a =18.8644(12) Å	α = 90°
	b = 10.3453 Å	β = 104.769(2)°
	c = 18.2109(10) Å	γ = 90°
Volume	3436.6(4) Å^3	
Z	4	
Density (calculated)	2.206 g cm^{-3}	
Absorption coefficient	7.465 mm^{-1}	
F(000)	2148.0	
2Θ range for data collection	4.466 to 53.46	
Reflections collected	134878	
Data/restraints/parameters	7296/1818/764	
Independent reflections	7296	
R_{int}	0.0938	
Crystal size	0.626 mm × 0.385 mm × 0.235 mm	
CCDC no.	2248987	

Table S2. Crystallographic properties of $[U^{III}\mathbf{2}(DMF)_2]I_3$

Chemical formula	$C_{22}H_{46}I_3N_4O_7U$	
Formula weight	1097.36	
R_1	0.0252	
wR_2	0.0668	
Goodness of Fit	1.031	
Largest residual peak/hole	1.83/–1.32	
Temperature	100.00 K	
Wavelength	0.71073 Å	
Crystal system	orthorhombic	
Space group	*Ibca*	
Unit cell dimensions	a = 14.2159(5) Å	α = 90°
	b = 14.8269(5) Å	β = 90°
	c = 31.2033(11) Å	γ = 90°
Volume	6577.0(4) $Å^3$	
Z	8	
Density (calculated)	2.216 g cm^{-3}	
Absorption coefficient	7.794 mm^{-1}	
F(000)	4104.0	
2Θ range for data collection	4.752 to 52.04	
Reflections collected	38026	
Data/restraints/parameters	3240/0/172	
Independent reflections	3240	
R_{int}	0.0474	
Crystal size	0.06 mm × 0.06 mm × 0.06 mm	
CCDC no.	2248985	

Table S3. Crystallographic properties of [U^{III}**2**$(CH_3CN)_2$]I_3

Chemical formula	$C_{20}H_{38}I_3N_4O_5U$	
Formula weight	1033.27	
R_1	0.0254	
wR_2	0.0635	
Goodness of Fit	1.030	
Largest residual peak/hole	1.93/–1.13	
Temperature	100 K	
Wavelength	0.71073 Å	
Crystal system	Orthorhombic	
Space group	*Ibca*	
Unit cell dimensions	a =13.9235(4) Å	α = 90°
	b = 14.4448(3) Å	β = 90°
	c = 30.0718(9) Å	γ = 90°
Volume	6048.1(3) $Å^3$	
Z	8	
Density (calculated)	2.270 g cm^{-3}	
Absorption coefficient	8.463 mm^{-1}	
F(000)	3816.0	
2Θ range for data collection	4.884 to 53.45	
Reflections collected	37598	
Data/restraints/parameters	3215/90/168	
Independent reflections	3215	
R_{int}	0.0536	
Crystal size	0.1 mm × 0.04 mm × 0.04 mm	
CCDC no.	2248986	

***SHAPE* Analysis**

Table S4: $[U^{III}\mathbf{1}(DMF)_2]I_3$

Shape	Shape code	Point group	CShM
Decagon	DP-10	D_{10h}	35.34052
Enneagonal pyramid	EPY-10	C_{9v}	23.49005
Octagonal bipyramid	OBPY-10	D_{8h}	18.64698
Pentagonal prism	PPR-10	D_{5h}	11.88054
Pentagonal antiprism	PAPR-10	D_{5d}	9.77952
Bicapped cube J15	JBCCU-10	D_{4h}	14.23529
Bicapped square antiprism J17	JBCSAPR-10	D_{4d}	5.46596
Metabidiminished icosahedron J62	JMBIC-10	C_{2v}	5.65646
Augmented tridiminished icosahedron J64	JATDI-10	C_{3v}	16.64308
Sphenocorona J87	JSPC-10	C_{2v}	4.65835
Staggered Dodecahedron (2:6:2)	SDD-10	D_2	5.79346
Tetradecahedron (2:6:2)	TD-10	C_{2v}	6.23016
Hexadecahedron (2:6:2) or (1:4:4:1)	HD-10	D_{4h}	11.90867

Table S5: $[U^{III}\mathbf{2}(CH_3CN)_2]I_3$

Shape	Shape code	Point group	CShM
Enneagon	EP-9	D_{9h}	34.74782
Octagonal pyramid	OPY-9	C_{8v}	25.74663
Heptagonal bipyramid	HBPY-9	D_{7h}	12.60957
Johnson triangular cupola J3	JTC-9	C_{3v}	17.15297
Capped cube J8	JCCU-9	C_{4v}	4.64799
Spherical-relaxed capped cube	CCU-9	C_{4v}	3.39415
Capped square antiprism J10	JCSAPR-9	C_{4v}	9.23594
Spherical capped square antiprism	CSAPR-9	C_{4v}	7.98444
Tricapped trigonal prism J51	JTCTPR-9	D_{3h}	9.78317
Spherical tricapped trigonal prism	TCTPR-9	D_{3h}	9.09181
Tridiminished icosahedron J63	JTDIC-9	C_{3v}	12.72477
Hula-hoop	HH-9	C_{2v}	4.68641
Muffin	MFF-9	C_s	5.99667

Table S6: [U^{III}2($DMF)_2]I_3$

Shape	Shape code	Point group	CShM
Enneagon	EP-9	D_{9h}	34.48255
Octagonal pyramid	OPY-9	C_{8v}	24.41299
Heptagonal bipyramid	HBPY-9	D_{7h}	13.21402
Johnson triangular cupola J3	JTC-9	C_{3v}	16.86874
Capped cube J8	JCCU-9	C_{4v}	4.98985
Spherical-relaxed capped cube	CCU-9	C_{4v}	3.81427
Capped square antiprism J10	JCSAPR-9	C_{4v}	7.83377
Spherical capped square antiprism	CSAPR-9	C_{4v}	6.81463
Tricapped trigonal prism J51	JTCTPR-9	D_{3h}	8.49939
Spherical tricapped trigonal prism	TCTPR-9	D_{3h}	7.73924
Tridiminished icosahedron J63	JTDIC-9	C_{3v}	12.14417
Hula-hoop	HH-9	C_{2v}	4.85224
Muffin	MFF-9	C_s	4.97180

Table S7: [U^{III}1(I)($CH_3CN)]I_2$*

Shape	Shape code	Point group	CShM
Decagon	DP-10	D_{10h}	36.41763
Enneagonal pyramid	EPY-10	C_{9v}	24.33195
Octagonal bipyramid	OBPY-10	D_{8h}	19.59560
Pentagonal prism	PPR-10	D_{5h}	13.24703
Pentagonal antiprism	PAPR-10	D_{5d}	10.31513
Bicapped cube J15	JBCCU-10	D_{4h}	15.54988
Bicapped square antiprism J17	JBCSAPR-10	D_{4d}	9.08357
Metabidiminished icosahedron J62	JMBIC-10	C_{2v}	9.10878
Augmented tridiminished icosahedron J64	JATDI-10	C_{3v}	18.27942
Sphenocorona J87	JSPC-10	C_{2v}	7.95305
Staggered Dodecahedron (2:6:2)	SDD-10	D_2	8.41509
Tetradecahedron (2:6:2)	TD-10	C_{2v}	9.15793
Hexadecahedron (2:6:2) or (1:4:4:1)	HD-10	D_{4h}	13.32313

***** D. N. Huh, J. M. Barlow, S. R. Ciccone, J. W. Ziller, J. Y. Yang and W. J. Evans, *Inorg. Chem.*, 2020, **59**, 17077–17083.

Cyclic voltammetry

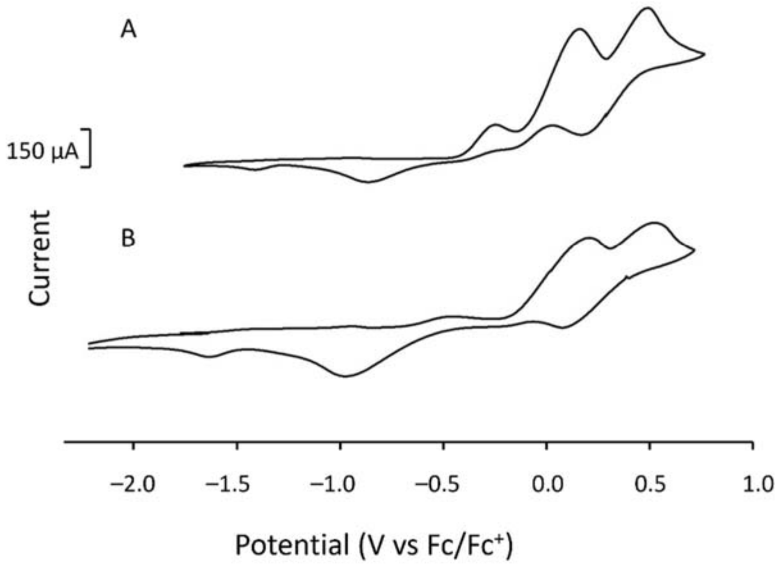

Figure S1. Cyclic voltammogram of (A) [U^{III}**1**]I_3 and (B) [U^{III}**2**]I_3 in CH_3CN [scan rate = 300 mV s^{-1}, and initial potential = –1.0 and –1.5 (V vs Ag/AgCl) for (A) and (B), respectively].

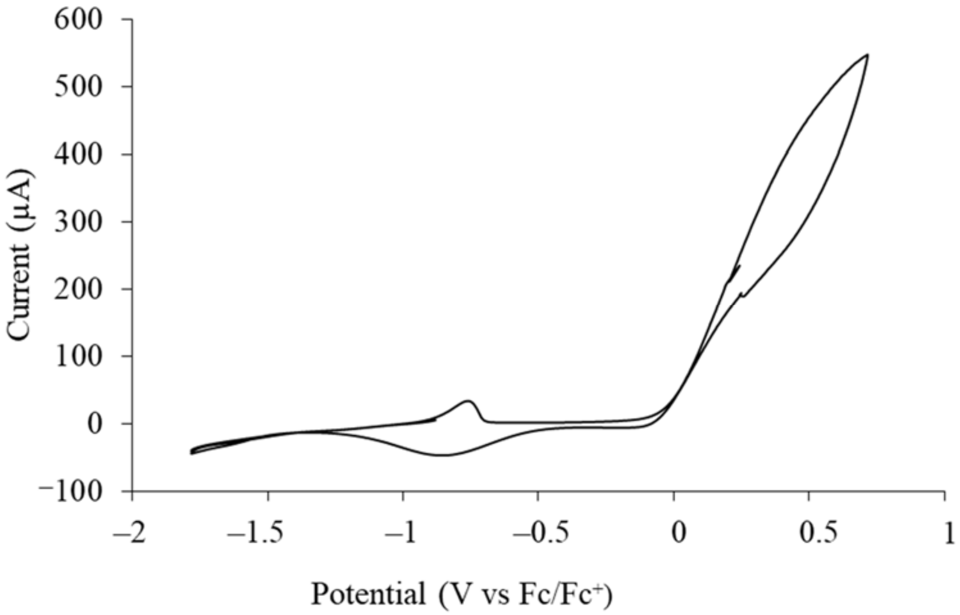

Figure S2. Cyclic voltammogram of UI_3(1,4-dioxane)$_{1.5}$ in acetonitrile [scan rate = 100 mV s^{-1}, and initial potential = –1.0 (V vs Ag/AgCl)].

Electronic absorption spectroscopy

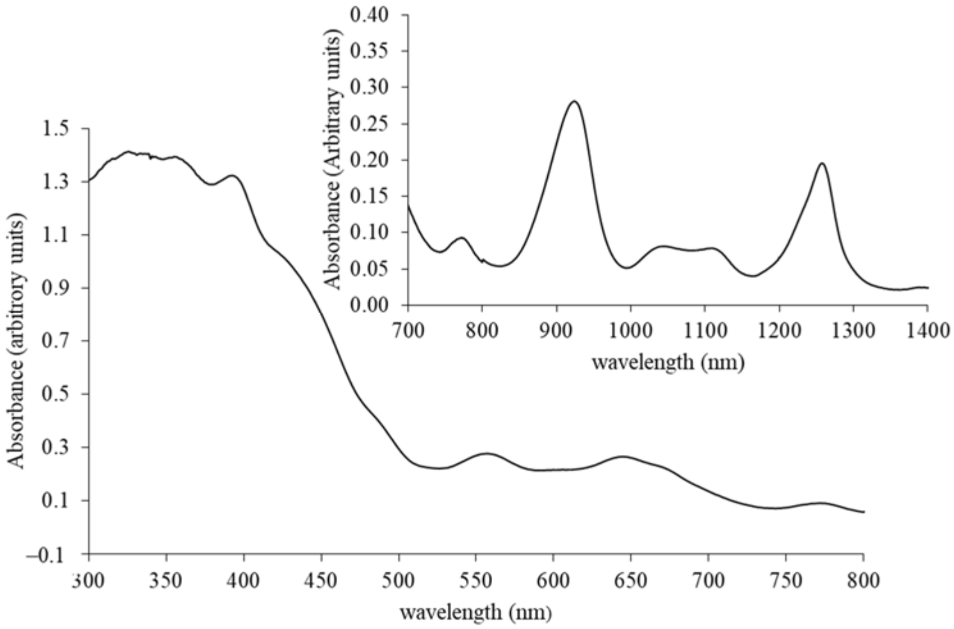

Figure S3. Electronic absorption spectra for $UI_3(1,4\text{-dioxane})_{1.5}$ in acetonitrile
